# Five doses of the mRNA vaccination potentially suppress ancestral-strain stimulated SARS-CoV2-specific cellular immunity: a cohort study from the Fukushima vaccination community survey, Japan

**DOI:** 10.3389/fimmu.2023.1240425

**Published:** 2023-08-16

**Authors:** Yuta Tani, Morihito Takita, Masatoshi Wakui, Hiroaki Saito, Takamitsu Nishiuchi, Tianchen Zhao, Chika Yamamoto, Takeshi Kawamura, Akira Sugiyama, Aya Nakayama, Yudai Kaneko, Tatsuhiko Kodama, Ryuzaburo Shinaha, Masaharu Tsubokura

**Affiliations:** ^1^ Medical Governance Research Institute, Tokyo, Japan; ^2^ Department of Radiation Health Management, Fukushima Medical University, Fukushima, Japan; ^3^ Department of Laboratory Medicine, Keio University School of Medicine, Tokyo, Japan; ^4^ Department of Internal Medicine, Soma Central Hospital, Fukushima, Japan; ^5^ Proteomics Laboratory, Isotope Science Center, The University of Tokyo, Tokyo, Japan; ^6^ Laboratory for Systems Biology and Medicine, Research Center for Advanced Science and Technology, The University of Tokyo, Tokyo, Japan; ^7^ Medical and Biological Laboratories Co., Ltd, Tokyo, Japan

**Keywords:** SARS-CoV2, vaccination, cellular immunity, immune imprinting, dialysis patient, vulnerable population

## Abstract

The bivalent mRNA vaccine is recommended to address coronavirus disease variants, with additional doses suggested for high-risk groups. However, the effectiveness, optimal frequency, and number of doses remain uncertain. In this study, we examined the long-term cellular and humoral immune responses following the fifth administration of the mRNA severe acute respiratory syndrome coronavirus 2 (SARS-CoV-2) vaccine in patients undergoing hemodialysis. To our knowledge, this is the first study to monitor long-term data on humoral and cellular immunity dynamics in high-risk populations after five doses of mRNA vaccination, including the bivalent mRNA vaccine. Whereas most patients maintained humoral immunity throughout the observation period, we observed reduced cellular immune reactivity as measured by the ancestral-strain-stimulated ELISpot assay in a subset of patients. Half of the individuals (50%; 14/28) maintained cellular immunity three months after the fifth dose, despite acquiring humoral immunity. The absence of a relationship between positive controls and T-Spot reactivity suggests that these immune alterations were specific to SARS-CoV-2. In multivariable analysis, participants aged ≥70 years showed a marginally significant lower likelihood of having reactive results. Notably, among the 14 individuals who received heterologous vaccines, 13 successfully acquired cellular immunity, supporting the effectiveness of this administration strategy. These findings provide valuable insights for future vaccination strategies in vulnerable populations. However, further research is needed to evaluate the involvement of immune tolerance and exhaustion through repeated vaccination to optimize immunization strategies.

## 1 Introduction

Broad application of the bivalent mRNA vaccine for Severe Acute Respiratory Syndrome Coronavirus 2 (SARS-CoV-2) is recommended for enhanced coverage and protection across the population (1). To provide enhanced protection against VoCs, the Centers for Disease Control and Prevention (CDC) recommends additional bivalent mRNA vaccine doses primarily for high-risk groups such as older individuals, patients undergoing dialysis, and those with moderate to severe immunodeficiency (2). However, no consensus on the effectiveness (3), optimal vaccination frequency, and number of doses (4) is established for the bivalent mRNA vaccine. Therefore, continuous evaluation of the effectiveness of the bivalent mRNA vaccine remains crucial even after the end of the pandemic (5).

Cellular immunity is pivotal in preventing the severe form of SARS-CoV-2 infection (6–8) due to the evasion of VoCs from neutralizing antibody recognition. Despite repeated vaccinations, specific vulnerable populations struggle to acquire sufficient immunity (7, 9–11). Thus, monitoring cellular immunity in high-risk groups is essential (12–14). However, limited comprehensive and long-term monitoring data is available to understand the humoral and cellular immunity dynamics in high-risk populations following the administration of the five-dose mRNA vaccination regimen, which includes the bivalent mRNA vaccine.

As of May 2023, the CDC’s guideline recommends that healthy individuals aged 6 years and older who are unvaccinated or previously given monovalent vaccine doses alone should receive a bivalent mRNA vaccine dose. The guideline also suggests an additional bivalent mRNA vaccine administration for individuals aged 65 years and older (2). As a prioritized group for vaccination, patients undergoing hemodialysis who are considered high-risk individuals are encouraged to receive additional vaccine doses (15). In Japan, administering the bivalent mRNA vaccine to older individuals and those with underlying high-risk medical conditions commenced in September 2022 (16). Since September 2021, we have been prospectively assessing humoral and cellular immunity in more than 2,500 residents and healthcare workers of the Soma, Minami-Soma, and Hirata villages in the Fukushima Prefecture in Japan (Fukushima vaccination cohort). In addition to humoral and cellular immunity data, we have obtained detailed profiling data from all participating individuals, including medical history and medication. Hence, the Fukushima cohort provided a valuable and distinct database (17–23), allowing us to assess the long-term dynamics of humoral and cellular immunity and analyze the characteristics of vulnerable populations.

Multiple methodologies are available to evaluate the SARS-CoV-2-specific T-cell immune memory by detecting cytokine production, especially for interferon-gamma (IFN-γ) release in antigen-stimulated short-term cultures *in vitro*, such as the enzyme-linked immunospot (ELISpot) assay, QuantiFERON assay, and analysis of intracellular expression by flow cytometry. We previously focused on dialysis patients as a high-risk group within the Fukushima cohort and reported the acquisition of humoral and cellular immunity after administering the third mRNA vaccine dose (24). Expanding on the previous research, we present the outcomes of a prolonged assessment, exploring both humoral and cellular immune responses after administering the fifth mRNA vaccine dose to the same dialysis patient population. In this study, we implemented the T-SPOT.COVID test, a standardized ELISpot IFN-γ release assay.

## 2 Materials and methods

### 2.1 Vaccination schedules, participant eligibility, and sample collection

**Figure 1:**
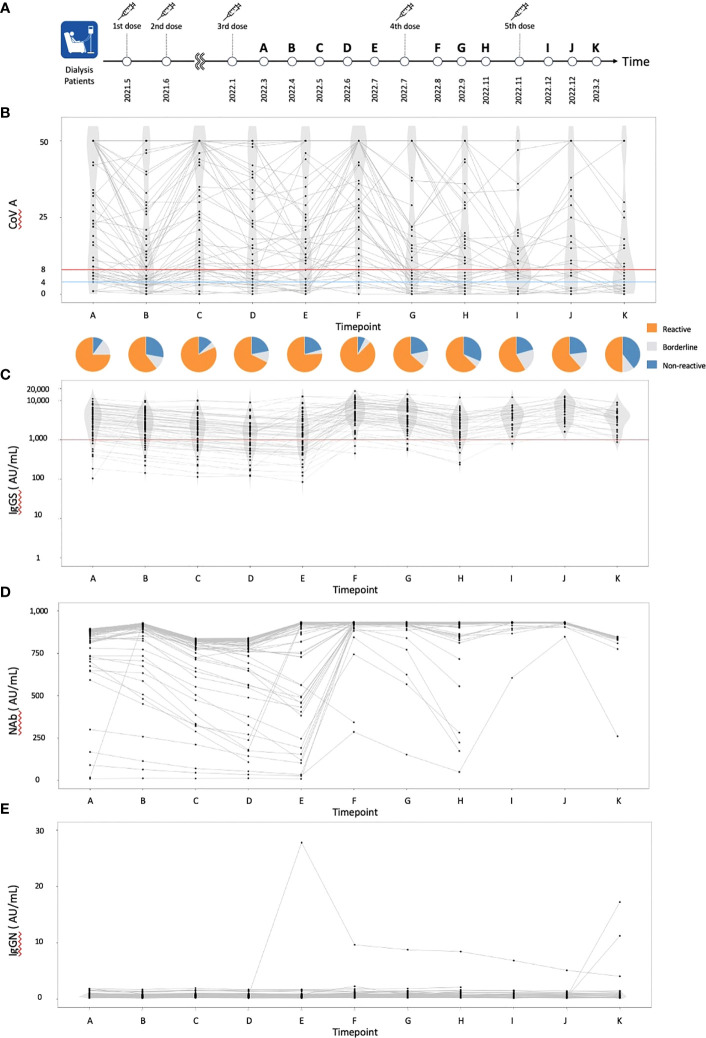
The dynamics of humoral and cellular immunity. **(A)** The timing of vaccinations and blood collections, **(B)** The dynamics of T-SPOT.COVID COV(A) results, **(C)** IgG(S) levels, **(D)** NAb titers, and **(E)** IgG against the nucleocapsid protein (IgG(N)) levels. In **(B)**, spots ≥50 are scored as 50. Spots ≤4, 5–7, and ≥8 were considered nonreactive, borderline, and reactive, respectively. Blue and red lines indicate the limit of detection as four spots and the limit of sensitivity as eight spots, respectively.

Study participants were recruited from patients undergoing dialysis in the Soma Central Hospital (Soma, Fukushima Prefecture, Japan) as a part of the Fukushima vaccination cohort study (24). They received the first, second, and fourth BNT162b2 vaccine doses (Pfizer/BioNTech, New York, NY, USA), the third dose with either BNT162b2 (Pfizer-BioNTech) or mRNA1273 (Moderna, Cambridge, UK), and the fifth dose with Comirnaty Bivalent Original/Omicron BA.4/5 (Pfizer-BioNTech). **Figure 1A** and **Supplementary Table 1** show the vaccination and blood collection timing. The first to fifth doses were administered in May 2021, June 2021, January 2022, July 2022, and November 2022, respectively. Peripheral blood collection (11 mL) was performed at the Some Central Hospital. The whole blood and serum samples were sent to the University of Tokyo (Tokyo, Japan) to measure SARS-CoV-2-specific antibodies and cellular immunity. Age, sex, days between vaccination and blood collection, vaccine type, smoking and drinking habits, and comorbidities were retrieved from a paper-based questionnaire.

### 2.2 Measurement of SARS-CoV-2-specific antibodies

The SARS-CoV-2-specific IgG (i.e., IgG(S)) and neutralizing activity (NAb) were measured as indicators of humoral immune status after vaccination. Chemiluminescent immunoassays were used using iFlash 3000 (YHLO Biotech, Shenzhen, China) and iFlash-2019-nCoV series (YHLO Biotech) reagents. All testing processes followed the official guidelines. Quality checks were conducted daily before measurements.

### 2.3 Measurement of cellular immunity

Peripheral blood was collected for the T-SPOT.COVID test (Oxford Immunotec, Abingdon, Cambridge, UK), a standardized ELISpot IFN-γ release assay. The blood specimens were processed and analyzed according to the manufacturer’s instructions. The samples were drawn into lithium heparin tubes and subsequently shipped to LSI Medience Corporation (Tokyo, Japan) in temperature-regulated boxes. This shipping process occurred overnight to ensure timely analysis. Next, the T-Cell Xtend reagent (Oxford Immunotec) was added to samples, and peripheral blood mononuclear cells were isolated using density gradient centrifugation. The cells were then washed, counted, and distributed at a density of 250,000 ± 50,000 cells/well for four different wells of a 96-well plate. We did not measure the viability in this assay. However, we ensured a consistent cell count of 250,000 ± 50,000 by measuring the white blood cell count using a hematology analyzer before conducting the test. Each well contained an optimized antigen pool containing the SARS-CoV-2 structural protein to stimulate T-cells *in vitro* and induce IFN-γ production. The IFN-γ released from the cells was captured by antibodies coated at the bottom of the wells. After 16–20 h incubation, alkaline phosphatase (AP)-conjugated secondary antibodies were added to bind to IFN-γ in the solid phase. Subsequently, substrates against AP were added, and the reaction displayed IFN-γ-producing spots. Along with the negative and positive controls (phytohemagglutinin [PHA] stimulation), SARS-CoV-2 spike antigen (COV(A)), and SARS-CoV-2 nucleocapsid antigen, four wells were used for each sample. The peptides were 15-mer peptides with 11 overlapping amino acids, and the pool comprised 253 peptides for antigen stimulation. The peptides were designed to be presented by major histocompatibility complex (MHC) class I or class II molecules, with MHC class I activating CD8+ T-cells and MHC class II activating CD4+ T-cells. The peptide pools are designed to encompass overlapping sequences across the entirety of SARS-CoV-2 proteins, potentially exhibiting some overlap with other coronaviruses, but predominantly containing a significant number of conserved epitopes shared among all strains of SARS-CoV-2. The results were interpreted by counting the spots in each well and subtracting the number of spots in the negative control as the background from the number of spots in the wells stimulated with the antigen. We utilized S6 TATC Entry Analyzer (CTL Corporation, Cleveland, Ohio, USA) as the ELISpot reader. The test was considered invalid if the number of spots in the negative control was >10. Following the manufacturer’s recommendations and criteria for spot-counting, four and eight spots were defined as the limits of detection and sensitivity. The results were then categorized based on the spot count: ‘nonreactive’ for four or fewer spots, ‘borderline’ for 5-7 spots, and ‘reactive’ for more than 8 spots. False positives may result from incorrect procedures, including improper blood sample collection or mishandling of specimens, as well as from prior exposure to SARS-CoV-1 and other closely related coronaviruses.

### 2.4 Statistical analysis

A descriptive analysis was conducted, and categorical variables were summarized as median (interquartile range) and numbers (percentages). The univariate and multivariate logistic regression analyses were performed to determine factors associated with T-SPOT.COVID COV(A) reactivity. For exploratory analysis 1, we used patient characteristics (age, sex, vaccine type, drinking habits, smoking, comorbidities, and IgG(S)) as explanatory variables. For exploratory analysis 2, we used age and levels of IgG(S), Nab, and the positive control at Timepoint A, approximately two months after third administration (**Supplementary Table 1**), as the explanatory variable. A repeated-measurement two-way ANOVA analysis was performed using the variables timepoint and reactive vs. nonreactive/borderline to assess the time-series results of the positive control. A two-sided *p*-value of <0.05 and <0.1 was considered statistically and marginally significant, respectively. The IBM SPSS Statistics (IBM ver. 28.0.1.0) software, R software (version 4.1.0, http://www.R-project.org), R package ggplot2 (version 3.3), R package magrittr (version 2.0.3), R package tidyr (version 1.3.0), R package dplyr (version 1.1.2), R package ggplot2 (version 0.4), and RStudio (Positive Software ver. 2023.03.1 + 446) were used for all analysis and figures.

## 3 Results

### 3.1 Humoral and cellular immunity dynamics in all participants

A total of 61 individuals participated in this study (**Table 1**). The median age was 70 years, with 55 individuals (90.0%) having hypertension, 30 (49.2%) having diabetes mellitus, and 7 (11.5%) having dyslipidemia. Sixteen individuals (26.2%) received a heterologous booster with mRNA-1273 for their third dose. The dynamics of T-SPOT.COVID COV(A), IgG(S), Nab titers, and the nucleocapsid protein (IgG(N)) levels results are shown in **Figures 1B–E**, respectively. The proportion of individuals reactive to COV(A) was 75.4% (46/61) at timepoint A but increased to 87.5% (49/56) at timepoint F following the fourth dose, 58.6% (17/29) at timepoint I after the fifth dose, 63.0% (17/27) at timepoint J, and 50.0% (14/28) at timepoint K. The proportion of individuals reactive to COV(A) decreased after timepoint G, two months after the fourth dose (**Figure 1B**). In contrast, the proportions of individuals positive for IgG(S) and NAb were consistently high across all time points. The proportion of individuals with IgG(S) levels ≥1000 AU/ml was 85.2% (52/61) at timepoint A, which increased to 96.4% (54/56) at timepoint F following the fourth dose, 96.6% (28/29) at timepoint I after the fifth dose, 100.0% (27/27) at timepoint J, and 96.4% (27/28) at timepoint K (**Figure 1C**). Similarly, the proportion of individuals with NAb titers ≥500 AU/ml was 93.4% (57/61) at timepoint A, which increased to 96.4% (54/56) at timepoint F following the fourth dose, 100.0% (29/29) at timepoint I after the fifth dose, 100.0% (27/27) at timepoint J, and 96.4% (27/28) at timepoint K (**Figure 1D**). Throughout the entire observation period, only three individuals had IgG(N) levels ≥10 AU/mL (**Figure 1E**).

**Table 1:** Participant characteristics.

Variables	N = 61
Age	70 [64–81]
< 70 years old	27 (44.3)
≥ 70 years old	34 (55.7)
Sex - Female	18 (29.5)
Vaccine type – heterologous-booster at the third dose	16 (26.2)
Smoking	6 (9.8)
Drinking habits	17 (27.9)
Comorbidities	
Hypertension	55 (90.2)
Diabetes mellitus	30 (49.2)
Dyslipidemia	7 (11.5)

Median [interquartile] or number (percentage) are shown for continuous or categorical variables.

### 3.2 Humoral and cellular immunity dynamics in the complete data cohort

**Figure 2:**
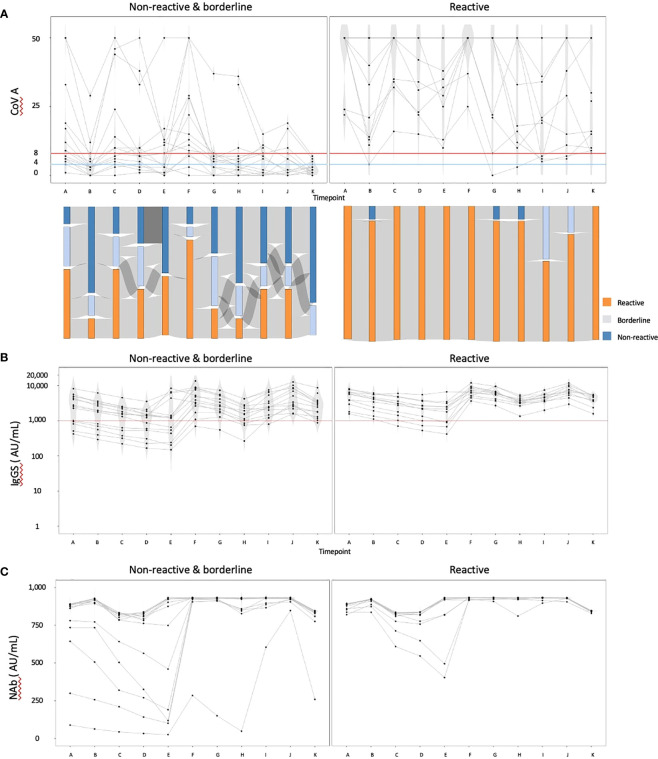
The dynamics of humoral and cellular immunity: comparison of the reactive and the nonreactive and borderline groups. We divided the cohort into two groups: the reactive group (n = 11) and the nonreactive and borderline group (n = 14), and illustrated the dynamics of humoral and cellular immunity. **(A)** The dynamics of T-SPOT.COVID COV(A) results, **(B)** IgG(S) levels, and **(C)** NAb titers. In **(A)**, spots ≥50 are scored as 50. Spots ≤4, 5–7, and ≥8 were considered nonreactive, borderline, and reactive, respectively. Blue and red lines indicate the limit of detection as four spots and the limit of sensitivity as eight spots, respectively.

We then focused on the cohort with data available at timepoint K and excluded individuals with IgG(N) ≥10 AU/mL (referred to as the ‘complete data cohort’). The complete data cohort comprised 25 individuals. The median age was 70 years, with 23 individuals (92.0%) having hypertension, 10 (40.0%) having diabetes mellitus, and three (12.0%) having dyslipidemia. Five individuals (20.0%) received a heterologous booster with mRNA-1273 for their third dose. Using the COV(A) results at timepoint K, we divided the cohort into two groups: the reactive group (n = 11) and the nonreactive and borderline group (n = 14). The humoral and cellular immunity dynamics are presented in **Figure 2**. The Sankey chart represents each group, excluding two individuals lacking data on some points. The proportions of individuals reactive to COV(A) at timepoints A and H were 100.0% (11/11) and 90.9% (10/11) in the reactive group, and 57.1% (8/14) and 21.4% (3/14) in the nonreactive and borderline group, respectively (**Figure 2A**). The proportions of individuals with IgG(S) ≥1000 AU/mL at timepoints A and H were both 100.0% (11/11) in the reactive group and 64.3% (9/14) and 76.9% (10/13) in the nonreactive and borderline group, respectively (**Figure 2B**). Similarly, the proportions of individuals with Nab ≥500 AU/mL at timepoints A and H were both 100.0% (11/11) in the reactive group and 85.7% (12/14) and 92.3% (12/13) in the nonreactive and borderline group, respectively (**Figure 2C**).

### 3.3 Exploratory analysis 1: patient characteristics associated with COV(A) reactivity after the fifth dose

The logistic regression analysis results using a complete data cohort for reactive COV(A) at timepoint K after the fifth dose are presented in **Table 2**. We excluded the hetero-booster and smoking as exploratory variables. In the multivariable analysis, participants aged ≥70 years (adjusted odds ratio (aOR): 0.087, 95% confidence interval (CI): 0.007–1.03, *p*-value: 0.052) showed a marginally significant lower likelihood of having reactive results. Notably, diabetes mellitus was not a significant factor in univariable and multivariable analyses. All five individuals who received a heterologous booster showed reactive results (OR: 2.89, 95% CI: 0.23–26.9, *p*-value: 0.41). For timepoint H after the fourth vaccine dose, logistic regression analysis was performed to determine reactive COV(A) in the 48 individuals (**Supplemental Table 2**). In the multivariable analysis, participants aged ≥70 years (aOR: 0.32, 95% CI: 0.087–1.19, *p*-value: 0.09) exhibited a marginally significant lower likelihood of having reactive results. Similar to the analysis at timepoint K, diabetes mellitus was not a significant factor in univariable and multivariable analyses. Among the 14 individuals who received a heterologous booster, 13 showed reactive results.

**Table 2:** Logistic regression analysis at timepoint K.

Variables	Reactive (n = 11)	Nonreactive and borderline (n = 14)	Univariable analysis		Multivariable analysis	
			OR (95% CI)	*p*-value	aOR (95% CI)	*p*-value
Age	66 [61–70]	80 [68–84]				
< 70 years old	7 (63.6)	3 (21.4)	1 (reference)	–	1 (reference)	–
≥ 70 years old	4 (36.4)	11 (78.6)	0.16 (0.027–0.92)	0.040	0.087 (0.007–1.03)	0.052
Sex - Women	4 (36.4)	5 (35.7)	1.03 (0.20–5.33)	0.97	1.52 (0.16–14.5)	0.71
Heterologous booster at the third dose	5 (45.5)	0 (0.0)	–	–	–	–
Drinking Habits *1 missing value	3 (27.3)	3 (21.4)	1.25 (0.20–8.0)	0.81	1.98 (0.18–21.7)	0.58
Smoking *1 missing value	1 (9.1)	0 (0.0)	–	–	–	–
Comorbidities						
Hypertension	10 (90.9)	13 (92.9)	0.77 (0.043–13.9)	0.86	–	–
Diabetes Mellitus	5 (45.5)	5 (35.7)	1.50 (0.30–7.53)	0.62	0.33 (0.025–4.25)	0.40
Dyslipidemia	2 (18.2)	1(7.1)	2.89 (0.23–36.9)	0.41	–	–

Median [interquartile] or number (percentage) are shown for continuous or categorical variables. 95% CI, 95% confidence interval; aOR, adjusted odds ratio; OR, odds ratio.

### 3.4 Exploratory analysis 2: Humoral and cellular immunity after the third dose associated with COV(A) reactivity after the fifth dose

We then conducted logistic regression analysis using humoral and cellular immunity at timepoint A as the explanatory variable to determine the COV(A) reactivity at timepoint K after the fifth dose. This analysis was done to understand how the initial humoral and cellular immune corrected with age would influence those after the repeated vaccinations of fifth dose. In the multivariable analysis (**Supplemental Table 3**), age (aOR: 0.86, 95% CI: 0.73–1.00, *p*-value: 0.051) and positive control (aOR: 0.99, 95% CI: 0.98–1.00, *p*-value: 0.056) were marginally significant factors. The time-dependent changes in the positive control are presented in **Supplementary Figure 1**, showing a consistent pattern over time, but no significant difference was observed between the reactive and nonreactive groups. A two-way ANOVA was performed to assess the significance of the positive control at different timepoints (point A to point K), revealing no significant effect of CoV(A) reactivity (reactive or nonreactive/borderline) (F-value: 0.713, *p*-value: 0.40), but a significant effect of timepoints was observed (F-value: 30.7, *p*-value <0.001). The interaction effect between the timepoints and COV(A) reactivity was insignificant, with an F-value of 0.185 and a *p*-value of 0.997.

### 3.5 The dynamics of humoral and cellular immunity in three patients with no reactive cellular immunity throughout the observation period

The dynamics of COV(A), the positive control, IgG(S), and NAb for the three individuals who did not show reactivity in COV(A) at any timepoints are shown in **Supplementary Figure 2**. The ages of these individuals were 78, 89, and 87 years, and all of them received a homologous booster. No significant medical histories other than hypertension among these individuals were reported. Patient 66 consistently maintained IgG(S) ≥1000 AU/mL and Nab ≥500 AU/mL. Patient 47 achieved IgG(S) ≥1000 AU/mL and Nab ≥500 AU/mL for the first time at timepoint F after the fourth dose and maintained these levels until timepoint K. Patient 44 achieved IgG(S) ≥1000 AU/mL and Nab ≥500 AU/mL for the first time at timepoint I after the fifth dose, but at timepoint K, the values were 1,014 and 259.86 AU/mL of IgG(S) and NAb, respectively.

### 3.6 Humoral and cellular immunity in three infected patients

The dynamics of COV(A), IgG(S), NAb, and IgG(N) for the three individuals with IgG(N) ≥10 AU/mL, suggesting SARS-CoV-2 infection, are shown in **Supplementary Figure 3**. Patient 49 was infected between timepoints D and E. No elevation was observed in the COV(A) score (50 at timepoints D and E), whereas slight increases were found in IgG(S) (8,915 and 12,681 AU/mL) and NAb (839 and 930 AU/mL) between the two timepoints. Patients 12 and 61 were infected between timepoints J and K. No COV(A) score increase was observed between the two timepoints (25 and 18 in patients 12 and 32, respectively, and 8 in patient 61). No significant changes in humoral and cellular immunity were observed before and after infection in the individuals.

## 4 Discussion

To the best of our knowledge, this is the first study to monitor long-term data on the dynamics of humoral and cellular immunity in high-risk populations following the administration of the five-dose mRNA vaccination regimen, including the bivalent mRNA vaccine. Although most participants acquired humoral immunity, 50% of individuals maintained cellular immunity three months after the fifth dose. Previously, in the same patients undergoing dialysis cohort, we reported that 71.4% (40/56) of individuals acquired cellular immunity two weeks after the third dose (24). In a healthy population, 64.3% (700/1089) of individuals acquired cellular immunity after the third dose (11). However, a consistent portion of the population remained unable to acquire cellular immunity even after the fifth dose, similar to the third dose. In a study using QuantiFERON, cellular immunity was observed in 50% (8/16) of individuals after the third dose (25). INF-γ, detected using T-SPOT.COVID and QuantiFERON, have been suggested to play a crucial role in SARS-CoV-2 infection and reinfection (8). Therefore, monitoring cellular immunity in high-risk populations is critical (13), and our results further support its significance.

The group that acquired cellular immunity after the third dose consistently maintained cellular immunity. This finding is consistent with previous reports stating that once cellular immunity is acquired, it can be maintained for several months (12, 26). In contrast, some groups could not acquire cellular immunity, regardless of receiving the fourth or fifth dose and acquiring efficient humoral immunity. Furthermore, we observed an increase in the proportion of individuals unable to acquire cellular immunity after the fifth dose. This finding might align with Gao et al.’s report using a mouse model, which showed a decrease in CD4+ and CD8+ T cell activity and an increase in Treg expression after the fifth and sixth doses of mRNA vaccination, suggesting the mechanism of immune tolerance (27). Since no relationship was observed between the positive controls and T-Spot reactiveness, these immune alternations are possibly specific to SARS-CoV-2. These specific immune alterations might involve the Tregs expression or the emergence of exhausted T cells. Multiple doses of mRNA vaccines are recommended for high-risk groups (28, 29). However, considering the potential for immune tolerance and exhaustion in cellular immunity after repeated mRNA vaccine administration (especially five or more doses), there might be an alternative strategy for SARS-CoV-2 immunization. The potential evasion of cellular immune responses to VoCs following the emergence of the Omicron variant (30) further emphasizes the need to re-evaluate vaccination strategies for high-risk groups. Thus, a booster with the monovalent vaccine targeting the latest VoC may be beneficial to avoid the suppression of cellular immune response observed in the part of our patients.

Aging was the only marginally significant factor associated with the inability to acquire cellular immunity. Previous reports have also suggested a significant correlation between aging and nonreactivity of cellular immunity (31, 32). Older individuals reportedly exhibit higher inefficiency in vaccine-induced spike-specific CD4+ T cell responses, limiting cellular immunity acquisition even after the second dose. Therefore, enhancing CD4+ T cell responses after the initial mRNA administration is critical in improving vaccine effectiveness among older people (33). Among the 14 individuals who received heterologous vaccines, 13 successfully acquired cellular immunity. This finding supports the effectiveness of the heterologous vaccine administration strategy (34–36). These results provide valuable data for considering future vaccination strategies for vulnerable populations. Other factors such as sex, alcohol consumption, smoking history, hypertension, diabetes mellitus, and dyslipidemia have been implicated in acquiring cellular immunity (31, 32). However, in this study, none of these factors were significant. Identifying specific groups unable to acquire cellular immunity based on HLA types remains a future research challenge (10).

Notably, 12% (3/25) of individuals could not acquire cellular immunity throughout the observation period. Therefore, their advanced age and homologous vaccination status should be considered. The presence of individuals who cannot acquire cellular immunity despite booster administration aligns with previous reports (33, 37). For such vulnerable populations, vaccination strategies that aim to induce a stronger T-cell response, such as the heterologous administration of mRNA vaccines (7), or considering the use of inactivated vaccines (38), may be worth exploring. The COV(A), IgG(S), and NAb dynamics in infected individuals are also intriguing. Given that the infections occurred after the emergence of the Omicron variant, the infection with post-Omicron VoCs may not significantly impact the humoral and cellular immunity specific to the Wuhan strain.

This study had several limitations. First, a lack of a control group hinders comparing results with healthy individuals or pre-vaccination values. Additionally, it prevents comparing the group receiving the fifth dose and those who did not receive the vaccine. Secondly, the proportion of patients with a history of SARS-CoV-2 infection was very small in the Fukushima vaccination cohort, making it challenging to evaluate the efficacy of repeated vaccination and the influences of natural SARS-CoV-2 infection on long-term cellular and humoral immunity. Thirdly, some bias existed owing to a high proportion of patients with hypertension and few participants with heterologous boosters. The nature of a single-site study might also cause unknown bias. Therefore, we could not justify the influence of such potent confounders in the study results. Fourthly, the limitation of IFN-gamma ELISPOT is its potential to miss IFN-gamma non-producing antigen-specific cells, such as those producing IL-4 or IL-17. Future research should combine flow cytometry and intracellular cytokine staining to address this issue and provide a more comprehensive evaluation of the immune response to specific antigens. Fifthly, the viability of PBMCs was not assessed in the TSPOT.COVID assay. Thus, the results might be influenced by the difference in viability during blood collection and subsequent cell processing. However, we made the best effort in the sample handling to minimize the viability-related issues and ensured consistent cell counts before the assay under standardized conditions. Lastly, the cellular immune response to other strains except the Wuhan strain is still unknown due to limited assay systems. Consequently, information regarding the effectiveness of the vaccines against VoCs cannot be inferred from this study.

## 5 Conclusion

In the present study, we elucidate the long-term results of cellular and humoral immune response after the fifth administration of the mRNA SARS-CoV-2 vaccination in patients undergoing hemodialysis. Humoral immunity to SARS-CoV-2 was maintained for the observation period in most patients; however, some patients had diminished cellular immune reactivity. Further study is needed to elucidate the efficacy of repeated vaccinations and optimize the immunization strategy.

## Data availability statement

The original contributions presented in the study are included in the article/**Supplementary Material**. Further inquiries can be directed to the corresponding author.

## Ethics statement

The studies involving humans were approved by the ethics committees of Hirata Central Hospital (number 2021-0611-1) and Fukushima Medical University School of Medicine (number 2021-116). The studies were conducted in accordance with the local legislation and institutional requirements. Written informed consent for participation in this study was provided by the participants’ legal guardians/next of kin.

## Author contributions

Concept and design, YT, MTa, and MTs. Acquisition, analysis, or interpretation of data, all authors. Drafting of the manuscript, YT, MTa, and MTs. Critical revision of the manuscript for important intellectual content, MW and HS. Statistical analysis, YT and MTa. Obtained funding, MTs. Administrative, technical, or material support, TZ, CY and TN. Supervision, MTs. All authors contributed to the article and approved the submitted version.
