# Supplementary material for: Five doses of the mRNA vaccination potentially suppress ancestral-strain stimulated SARS-CoV2-specific cellular immunity: a cohort study from the Fukushima vaccination community survey, Japan
Source: Front Immunol. 2023 Aug 16;14:1240425. doi: 10.3389/fimmu.2023.1240425 (PMC10469480; doi:10.3389/fimmu.2023.1240425)
Supplement: Supplementary file 1 [file DataSheet_1.docx]

Supplementary Material

Five doses of the mRNA vaccination potentially suppress ancestral-strain stimulated SARS-CoV2-specific cellular immunity: A cohort study from the Fukushima Vaccination Community Survey, Japan

**Yuta Tani^1^, Morihito Takita^1,2^, Masatoshi Wakui^3^, Hiroaki Saito^2,4^, Tianchen Zhao^2^, Chika Yamamoto^2^, Takeshi Kawamura^5,6^, Akira Sugiyama^5^, Aya Nakayama^5^, Yudai Kaneko^6,7^, Tetsuhiko Kodama^6^,** **Masaharu Tsubokura^1,2,4^***

^1^Medical Governance Research Institute, Tokyo, Japan

^2^Department of Radiation Health Management, Fukushima Medical University, Fukushima, Japan

^3^Department of Laboratory Medicine, Keio University School of Medicine, Tokyo, Japan

^4^Department of Internal Medicine, Soma Central Hospital, Fukushima, Japan.

^5^Proteomics Laboratory, Isotope Science Center, The University of Tokyo, Tokyo, Japan.

^6^Laboratory for Systems Biology and Medicine, Research Center for Advanced Science and Technology, The University of Tokyo, Tokyo, Japan.

^7^Medical and Biological Laboratories Co., Ltd, Tokyo, Japan.

*** Correspondence:**Masaharu Tsubokura
[tsubo-m@fmu.ac.jp](mailto:tsubo-m@fmu.ac.jp)

# Supplementary Data Legends

Supplementary Table 1. Dates of vaccinations and blood collections

Supplementary Table 2. Logistic regression analysis at timepoint H

Supplementary Table 3. Logistic regression analysis at timepoint K using data at timepoint A as explanatory variables

Supplementary Figure 1. The dynamics of T-SPOT.COVID phytohemagglutinin (PHA) results

Supplementary Figure 2 The dynamics of humoral and cellular immunity for the three individuals who did not show reactivity

Supplementary Figure 3 The dynamics of humoral and cellular immunity for the three individuals with IgG(N) ≥ 10 AU/mL, suggesting SARS-CoV-2 infection

# Supplementary Figures and Tables

**Supplementary Table 1. Detail data on vaccinations and blood collections**

| Timepoint | First dose | Second dose | Third dose | A | B | C | | D | E | Fourth dose | F | G | | H | Fifth dose | I | J | K |
| --- | --- | --- | --- | --- | --- | --- | --- | --- | --- | --- | --- | --- | --- | --- | --- | --- | --- | --- |
| Year | 2021 | | | | | | 2022 | | | | | | 2023 | | | | | |
| The earliest date (month/day) | 5/12 | 6/2 | 1/26 | 3/28 | 4/25 | 5/23 | | 6/20 | 7/20 | 7/8 | 8/22 | 9/21 | | 11/21 | 11/28 | 12/5 | 12/19 | 2/20 |
| The latest date  (month/day) | 6/22 | 7/13 | 2/25 | 4/14 | 5/12 | 5/24 | | 6/21 | 7/21 | 8/23 | 8/23 | 9/22 | | 11/22 | 11/29 | 12/6 | 12/20 | 2/21 |
| Days between the last vaccination and blood collection | - | - | - | 61  [40–61] | 89  [68–89] | 117  [96–117] | | 145  [124–145] | 175  [154–175] | - | 28  [28–28] | 58  [56–58] | | 119  [119–119] | - | 7  [7–7] | 21  [21–21] | 84  [84–94] |

Median [interquartile] is shown for days between the last vaccination and blood collection.

**Supplemental Table 2. Results of logistic regression analysis at timepoint H**

| Variables | Reactive  (n = 31) | Nonreactive and borderline (n = 17) | Univariable analysis | | Multivariable analysis | |
| --- | --- | --- | --- | --- | --- | --- |
|  |  |  | OR (95% CI) | *p*-value | aOR (95 % CI) | *p*-value |
| Age | 68 [64–74] | 80 [66–83] |  |  |  |  |
| < 70 years old | 17 (54.8) | 5 (29.4) | 1 (reference) | 0.096 | 1 (reference) | 0.090 |
| ≥ 70 years old | 14 (45.2) | 12 (70.6) | 0.34 (0.097–1.21) | 0.096 | 0.32 (0.087–1.19) | 0.090 |
| Sex - Female | 9 (29.0) | 5 (29.4) | 0.98 (0.27–3.6) | 0.98 | 0.83 (0.21–3.28) | 0.79 |
| Heterologous booster at the third dose | 13 (41.9) | 1 (5.9) | 11.6 (1.36–98.5) | 0.025 | - | - |
| Drinking habits | 13 (41.9) | 1 (5.9) | 11.6 (1.36—98.5) | 0.025 | - | - |
| Smoking | 5 (16.1) | 0 (0.0) | - | - | - | - |
| Comorbidities |  |  |  |  |  |  |
| Hypertension | 30 (96.8) | 15 (88.2) | 4.0 (0.34–47.7) | 0.27 | - | - |
| Diabetes mellitus | 15 (48.4) | 8 (47.1) | 1.06 (0.32–3.45) | 0.93 | 0.85 (0.24–3.00) | 0.81 |
| Dyslipidemia | 6 (16.1) | 1 (5.9) | 3.08 (0.33–28.8) | 0.32 | - | - |

Median [interquartile] or number (percentage) are shown for continuous or categorical variables. 95% CI, 95% confidence interval; aOR, adjusted odds ratio; OR, odds ratio.

**Supplemental Table 3. Logistic regression analysis at timepoint K using data at timepoint A as explanatory variables**

| Variables | Reactive  (n = 11) | Nonreactive and Borderline (n = 14) | Univariable analysis | | Multivariable analysis | |
| --- | --- | --- | --- | --- | --- | --- |
|  |  |  | OR (95% CI) | *p*-value | aOR (95% CI) | *p*-value |
| Age | 66 [61–70] | 79.5 [68–84] | 0.92 (0.84–1.00) | 0.048 | 0.86(0.73–1.00) | 0.051 |
| CoV(A) reactive | 11 (100) | 8 (57.1) | - | - | - | - |
| IgG(S) (1000AU/mL) | 5.67 [2.95–6.48] | 2.67 [0.91–4.96] | 1.42 (0.97–2.08) | 0.071 | 1.23(0.67–2.28) | 0.50 |
| N(Ab) (100AU/mL) | 8.85 [8.54–8.91] | 8.69 [7.12–8.89] | 3.25 (0.45–23.6) | 0.24 | 2.46(0.42–14.4) | 0.32 |
| Positive control | 323 [182–389] | 368 [245–524] | 1.00 (0.99–1.00) | 0.11 | 0.99(0.98–1.00) | 0.056 |

Median [interquartile] or number (percentage) are shown for continuous or categorical variables. 95% CI, 95 % confidence interval; aOR, adjusted odds ratio; OR, odds ratio. The positive control referred to the PHA-stimulated wells of T-SPOT.COVID assay.

**Supplementary Figure 1. The dynamics of T-spot COVID phytohemagglutinin results**


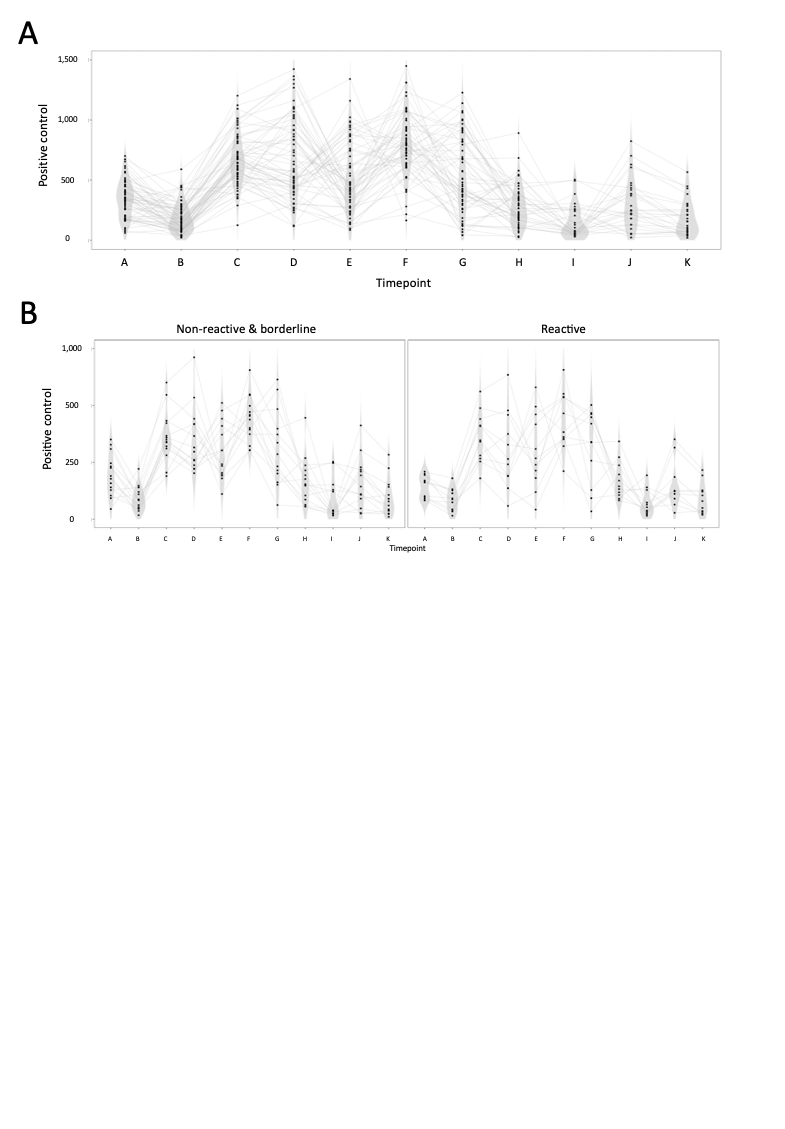


(A) The dynamics of T-SPOT.COVID phytohemagglutinin (PHA) results. We divided the cohort into two groups: the reactive group (n = 11) and the nonreactive and borderline group (n = 14), and (B) illustrated the dynamics of the T-SPOT.COVID PHA results.

**Supplementary Figure 2. The dynamics of humoral and cellular immunity for the three individuals who did not show reactivity**


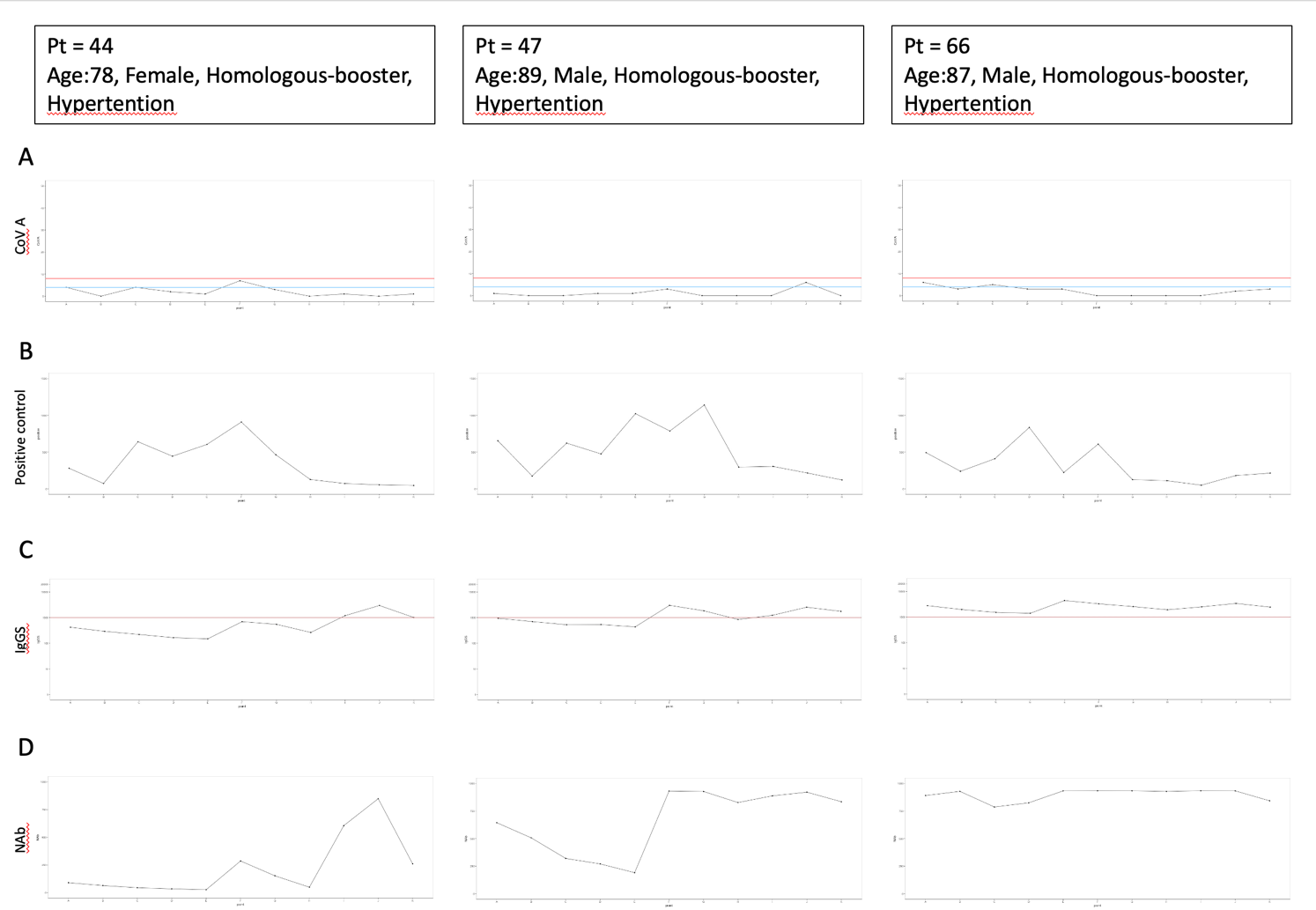


(**A**) The dynamics of T-SPOT.COVID COV(A) results, (**B**) T-SPOT.COVID phytohemagglutinin (PHA) results, (**C**) IgG(S) levels, and (**D**) NAb titers. In (**A**), spots ≥50 are scored as 50. Spots ≤4, 5–7, and ≥8 were considered nonreactive, borderline, and reactive, respectively.

**Supplementary Figure 3. The dynamics of humoral and cellular immunity for the three individuals with IgG(N) ≥ 10 AU/mL suggest SARS-CoV-2 infection**


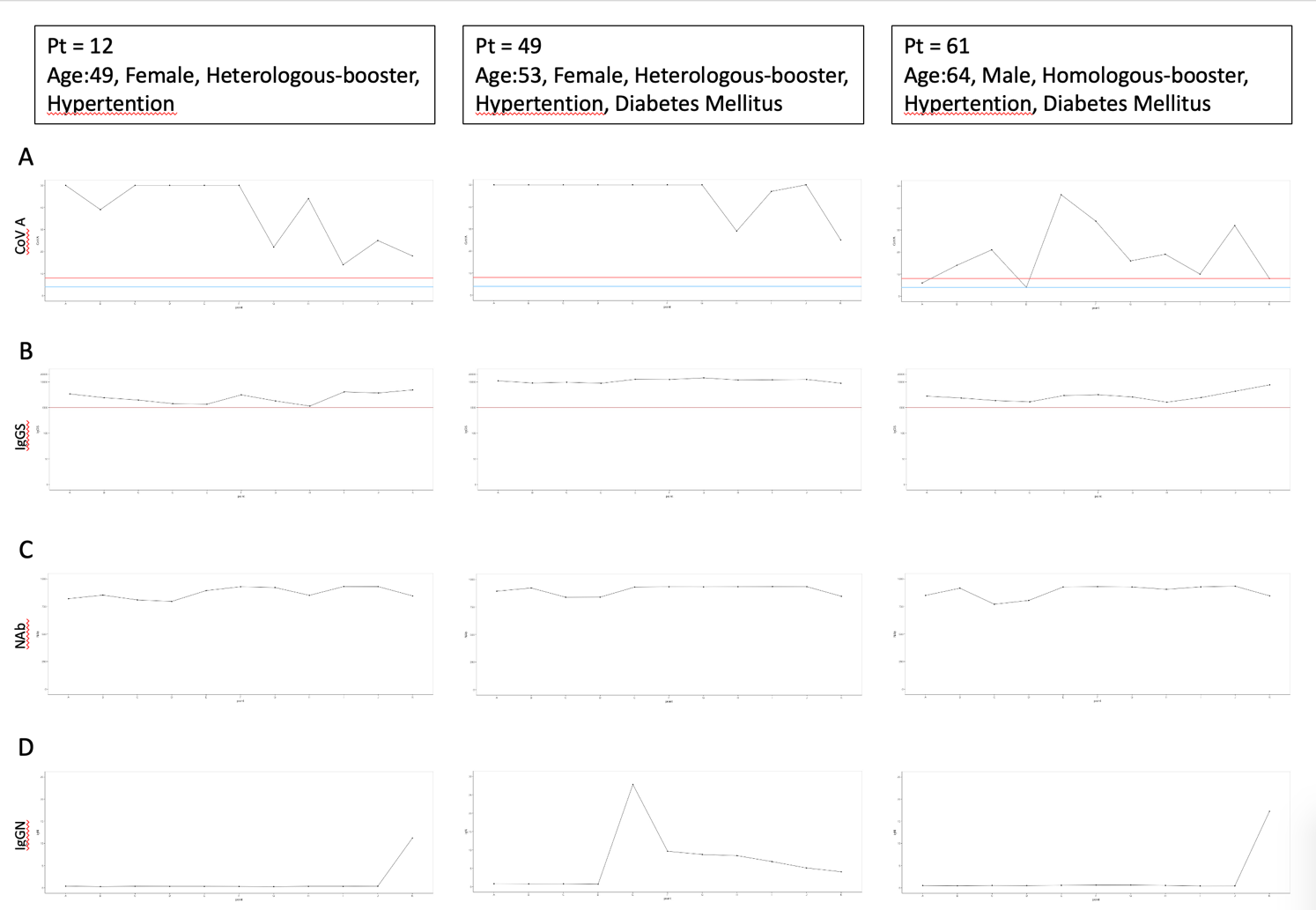


(**A**) The dynamics of T-SPOT.COVID COV(A) results, (**B**) IgG(S) levels, (**C**) NAb titers, and (**D**) IgG against the nucleocapsid protein (IgG(N)) levels. In (**A**), spots ≥50 are scored as 50. Spots ≤4, 5–7, and ≥8 were considered nonreactive, borderline, and reactive, respectively.
